# Tyrosine Kinase Inhibitors as Reversal Agents for ABC Transporter Mediated Drug Resistance

**DOI:** 10.3390/molecules190913848

**Published:** 2014-09-04

**Authors:** Nagaraju Anreddy, Pranav Gupta, Rishil J. Kathawala, Atish Patel, John N. D. Wurpel, Zhe-Sheng Chen

**Affiliations:** Department of Pharmaceutical Sciences, College of Pharmacy and Health Sciences, St. John’s University, Queens, NY 11439, USA; E-Mails: nagaraju.anreddy08@my.stjohns.edu (N.A.); pranav.gupta13@my.stjohns.edu (P.G.); rishil.kathawala10@my.stjohns.edu (R.J.K.); atishpatel268@gmail.com (A.P.); WURPELJ@stjohns.edu (J.N.D.W.)

**Keywords:** tyrosine kinase inhibitors, ABC transporters, chemotherapy, modulators, multidrug resistance

## Abstract

Tyrosine kinases (TKs) play an important role in pathways that regulate cancer cell proliferation, apoptosis, angiogenesis and metastasis. Aberrant activity of TKs has been implicated in several types of cancers. In recent years, tyrosine kinase inhibitors (TKIs) have been developed to interfere with the activity of deregulated kinases. These TKIs are remarkably effective in the treatment of various human cancers including head and neck, gastric, prostate and breast cancer and several types of leukemia. However, these TKIs are transported out of the cell by ATP-binding cassette (ABC) transporters, resulting in development of a characteristic drug resistance phenotype in cancer patients. Interestingly, some of these TKIs also inhibit the ABC transporter mediated multi drug resistance (MDR) thereby; enhancing the efficacy of conventional chemotherapeutic drugs. This review discusses the clinically relevant TKIs and their interaction with ABC drug transporters in modulating MDR.

## 1. Tyrosine Kinases and Their Role in Cancer

Tyrosine kinases (TKs), a large and diverse category of enzymes, can catalyze the transfer of a phosphate group from ATP to target proteins and play vital roles in cell signal transduction pathways that regulate cell proliferation, differentiation, anti-apoptotic signaling and programmed cell death [1]. These TKs can be categorized as receptor TKs (RTKs) and non-receptor TKs (NRTKs). The RTKs contain an extracellular ligand binding domain and an intracellular catalytic domain with intrinsic TK activity [2]. Binding of a ligand to these receptors results in activation of downstream effects including stimulation of other TKs, increasing the levels of intracellular calcium, and activation of serine/threonine kinases, phospholipase C and phosphatidylinositol-3’-kinase, which further leads to changes in gene expression [2]. NRTKs do not have transmembrane domains and are present in the cytosol, the nucleus, and at the inner surface of the plasma membrane. A multitude of intracellular signaling events, oligomerzation, autophosphorylation and transphosphorylation by other kinases are responsible for stimulation of NRTKs. In the absence of an inhibitor, NRTKs are able to be stimulated or recruited to transmembrane receptors, resulting in many possible downstream phosphorylation events [3]. Both RTKs and NRTKs are vital mediators of intracellular signal transduction pathways. Three cytoplasmic signaling pathways that are activated by RTKs playing pivotal roles in cell proliferation and homeostasis [4]. These include Ras/Raf MAPK pathway, the phosphoinositol 3-kinase/Akt (PI3K/Akt) pathway, and the JAK/STAT pathway. Any aberration in TK structure or function results in interruption of these signaling pathways, thereby disrupting a wide array of cellular functions which eventually results in increased cell proliferation and decreased apoptosis. This imbalance in cellular homeostasis results in initiation of the oncogenic transformation process, which further leads to cancer development or malignancy resulting in metastasis [5].

## 2. TKIs and Their Role in Cancer Chemotherapy

Atypical activity of TKs is controlled by inhibiting the catalytic activity of the kinases. A significant amount of work in this field has been done to discover small molecule inhibitors that can block the phosphorylation mediated by these kinases. Several classes of inhibitors have been developed that interfere with the activity of deregulated kinases at different levels. These can be generally classified into three categories: (i) Drugs that inhibit ATP binding to the ATP binding site of kinases, such as imatinib, nilotinib, and dasatinib (BCR-ABL kinase inhibitors) and gefitinib, erlotinib and lapatinib (epidermal growth factor receptor inhibitors); (ii) Drugs that inhibit allosteric sites (binding site for a particular kinase) required for the activation of kinases, allosteric inhibitors includes BIRB 796 (p38 inhibitor), BAY43-9006 (Raf inhibitor), and PD184352 (MAP kinase inhibitor). Owing to their specificity in binding to a particular kinase, these inhibitors are top ranked in terms of their kinase selectivity; (iii) Drugs that inhibit fusion of TKs by blocking their dimerization interrupt TK signaling through neutralization of ligands, block ligand binding or receptor internalization.

A substantial number of TKIs have been approved for cancer intervention or are presently being investigated in clinical trials [6]. These TKIs primarily inhibit or interfere with protein phosphorylation mediated cellular signaling pathways that control tumor cell growth by targeting various tyrosine kinases. However, multidrug resistance (MDR) not only affects the therapeutic potential of conventional antineoplastic agents, but also affects the function of TKIs in cancer chemotherapy. Undeniably, some ATP-binding cassette (ABC) transporters play a significant role in resistance to TKIs [7]. More importantly, some TKIs have also been shown to inhibit the drug efflux function of MDR-related ABC transporters and to circumvent the resistance of cancer cells to conventional chemotherapeutic drugs [8]. This review will focus on the small molecule TKIs used currently in cancer therapy and precisely their effect on ABC drug transporters mediated MDR.

## 3. Multidrug Resistance

**Figure 1:**
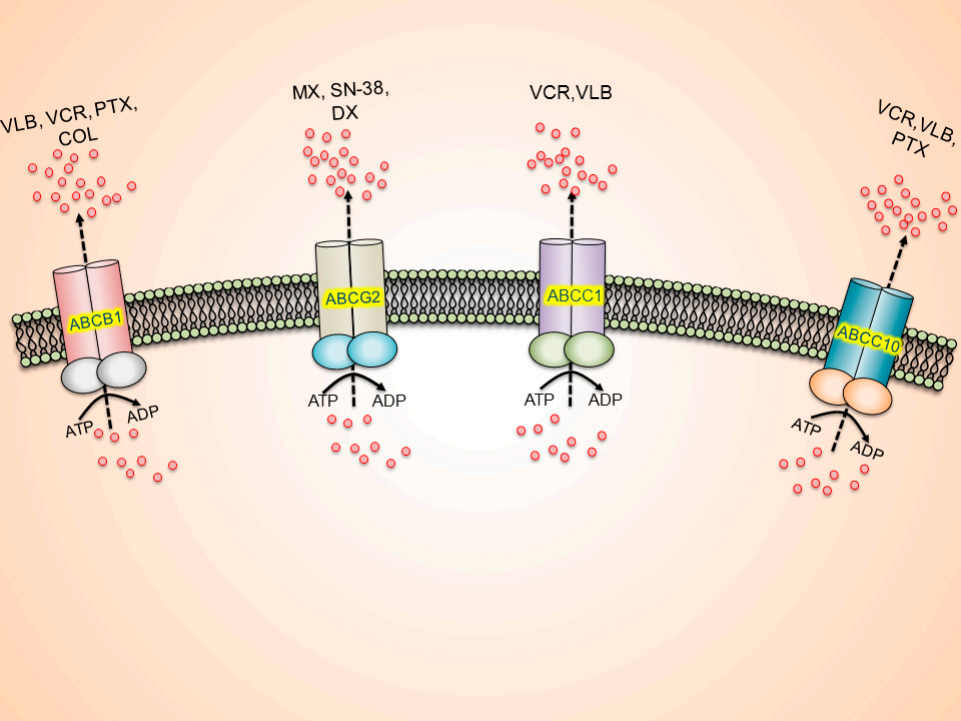
ABC drug transporter-mediated multi drug resistance (MDR).

The most common mechanisms that produce drug resistance in cancer cells include: (1) altered cell cycle check points; (2) induction of emergency response genes; (3) alterations in membrane lipids; (4) compartmentalization; (5) inhibition of apoptosis; (6) altered drug targets; (7) decreased uptake and (8) increased efflux of drugs [9,10,11,12,13]. In addition, one type of resistance that is highly problematic is MDR (Figure 1). MDR is a phenomenon whereby cancer cells become resistant to structurally and mechanistically distinct class of compounds [10,14,15].

One of the most common mechanisms that produce MDR in cancer cells is the presence of a family of specific transmembrane, energy dependent transporters known as ABC transporters. The ABC transporters are the most abundant transmembrane protein family encoded in the human genome [16,17]. ABC transporters “pump” drugs, such as anti-neoplastic drugs, out of the cells against a concentration gradient [18,19]. This was first reported by Dano whose research showed a decrease in the concentration gradient of daunorubicin in Erlich ascites cells by active extrusion [20]. Clearly, a broader understanding of the ABC family of transporters would represent a valuable strategy to surmount MDR in cancer cells. Currently, chemotherapy is a first-line approach for the treatment of most cancers [18,21,22,23,24,25,26]. However, even with the introduction of newer drugs and the application of different chemotherapeutic regimens, cancer cells can become resistant to antineoplastic drugs, thus, decreasing the likelihood of successful treatment [27,28,29].

### 3.1. ATP-Binding Cassette (ABC) Transporters

ABC transporters are a group of active transporter proteins that have diverse functions and ubiquitous presence in both prokaryotes and eukaryotes [18,30,31,32]. The ABC transporter efflux activity is driven by the energy derived from the hydrolysis of ATP to adenosine diphosphate (ADP) to transport substrates across the cellular membrane against a concentration gradient [19,33,34]. Till date, 48 members of the ABC transporter family have been isolated and identified [35,36]. The ABC transporter family is divided into seven subfamilies, ABCA through ABCG [35,37]. Structurally, ABC transporter proteins have two nucleotide binding domains (NBDs) and two transmembrane domains (TMDs) [18,38]. ABC transporters can be topologically classified based on the sequence of the NBDs, also known as ABC domains [14,16,39]. The NBDs are proteins consisting of conserved ABC (necessary for cellular function) that is responsible for binding and extruding physiological and xenobiotic substrates out of the cell. The NBDs, in addition to containing Walker A and B motifs, also contain an additional element, the signature (C) motif, found upstream to Walker B motif joining the Walker A and B motifs [40]. These Walker A and B motifs play a role in hydrolysis of ATP to ADP + Pi and energy coupling [41,42]. The NBD hydrolyzes ATP via ATPase enzyme and plays a crucial role in conferring MDR to various chemotherapeutic agents [17,28,31,43].

The majority of the members of the ABC transporter superfamily are full or complete transporters, *i.e.*, they have at least two NBDs and two TMDs [35]. There are some members, such as the ABCG subfamily, that are half transporters as they have only one NBD and TMD [44]. There is considerable evidence suggesting that two NBDs are required for normal transporter activity of ABC transporters. A study by Sauna *et al.* on ABCB1 (P-gp or MDR1) transporters showed that two ATPs are hydrolyzed in a stepwise process during the efflux of drugs by the ABCB1 transporter. The hydrolysis of first ATP by ATPase produces a structural modification of the TMDs that flips the inner leaf to the outer side of the cell membrane, leading to efflux of the drug from the cell [43,45]. The hydrolysis of the second ATP by ATPase restores the structure of the transporter to its original high affinity state, for the transport of other substrates [45,46].

Numerous studies indicate that ABC transporters are involved in efflux of xenobiotics out of the cell [47]. In addition, they play a role in transporting important substrates across extracellular and intracellular membranes, such as amino acids, cholesterol and its derivatives, sugars, vitamins, peptides, lipids, some important proteins, hydrophobic drugs and antibiotics [46,48,49,50,51].

ABC drug transporters increase the efflux of their substrate anti-cancer agents, resulting in reduced intracellular concentration of anti-cancer agents, thereby developing the MDR phenotype. ABCB1 substrates include vinblastine (VLB), vincristine (VCR), paclitaxel (PTX), and colchicine (COL). ABCG2 substrate includes mitoxantrone (MX), 7-ethyl-10-hydroxycamptothecin (SN-38) and doxorubicin (DX). ABCC1 substrate includes VCR and VLB. ABCC10 substrate includes VCR, VLB and PTX.

#### 3.1.1. ABCB1/P-glycoprotein (P-gp/MDR1)

ABCB1 has a molecular weight of 160-170-kDa and consists of two TMDs and two NBDs [28,52]. The ABCB1 is an apical membrane transporter that is present in the kidney, placenta, liver, adrenal glands, intestine and blood-brain barrier cells, where it functions to protect against xenobiotics and cellular toxicants [28,52,53]. The overexpression of ABCB1 confers significant resistance to a wide variety of compounds that are used in the chemotherapy of cancer [52,54,55]. The ABCB1 transporter is localized in the apical membrane of the small and large intestinal epithelium, in the syncytiotrophoblast of placenta, in the bile canalicular membrane of liver hepatocytes, at the apical membrane of renal proximal tubules and on the luminal surface of endothelial cells for brain capillaries and testes [56,57]. The cellular localization of ABCB1 allows it to mediate the efflux of exogenous and endogenous toxicants from cells into the urine and bile, thereby promoting their excretion from the body [47,53,58]. The overexpression of ABCB1 has been associated with various cancers, such as acute myeloid leukemia, childhood tumors, breast cancers, hematological malignancies and solid tumors to name a few [59,60,61,62]. The transporter is encoded in the *MDR1* gene [63,64]. Humans have two *MDR* genes, *MDR1* and *MDR2*, where MDR1*-*ABCB1 functions as a drug transporter and MDR2 ABCB1 as a phospholipid transporter [64,65].

#### 3.1.2. ABCC/Multidrug Resistance Proteins (MRPs)

The ABCC family consists of 13 subfamily members (ABCC1 to ABCC13). The ABCC transporters are present in various cancers, including lung (both small and non-small cell lung cancers) bladder and breast [66]. Of all the ABCC members, MRP10 (ABCC13) does not encode for a functional protein, *i.e.*, it does not play a role in mediating MDR and its physiological functions remains to be determined [67]. The MRP family can be further subdivided into two groups depending upon their structural topology. One group known as long MRPs consisting of MRP1 (ABCC1), MRP2 (ABCC2), MRP3 (ABCC3), MRP6 (ABCC6) and MRP7(ABCC10), have three TMDs and two NBDs, and the other group known as short MRPs consists of MRP4 (ABCC4), MRP5 (ABCC5), MRP8 (ABCC11) and MRP9 (ABCC12) that have two TMDs and two NBDs [27,68,69]. It should be noted that ABCC7 or CFTR (cystic fibrosis transmembrane regulator protein) is not involved in direct transport of substrates, but is a part of a chloride ion channel [66,67]. ABCC8 or SUR8 (sulfonylurea receptor 8) and ABCC9 or SUR9 (sulfonylurea receptor 9) are also involved in the regulation of potassium ion channels [46,70,71]. It has been reported that ABCC1, ABCC2, ABCC3, ABCC4, ABCC5 and ABCC6 play a significant role in MDR mediated non-small cell lung cancer (NSCLC), breast cancer, prostate cancer, colorectal cancer, ovarian cancer and acute lymphoblastic leukemia (ALL) [67,68]. In addition, recent findings have shown that ABCC10 (MRP7), ABCC11 (MRP8) and ABCC12 (MRP9) are functional transporters involved in conferring resistance to various anticancer drugs as shown in Table 1 [67,72].

ABCC1 is a 190-kDa protein that was first discovered in DX resistant HL60/Adr and H69AR cell lines [67,69,73]. Its substrate profile is similar to that of ABCB1, except that it has low affinity for taxanes (e.g. PTX, docetaxel) [72]. The substrates of the ABCC1 transporter include negatively charged lipophilic compounds, whereas ABCB1 has a high affinity for neutral and positively charged compounds [74,75].

The ABCC10/MRP7 is a 171-kDa protein that contains three TMDs and two NBDs and it belongs to the class of long MRPs, such as ABCC1, ABCC2, ABCC3, ABCC6 [76,77,78]. Hopper *et al.* using reverse transcription-PCR analysis, reported a low level of *MRP7* transcript expression in the skin, testes, spleen, stomach, colon, kidney, heart and brain [67,68,69,70,71,72,73,74,75,76,77,78,79]. However, *MRP7* transcripts were difficult to detect by Northern blot analysis, suggesting that it has a low level of expression in many tissues. However, it has been reported that *MRP7* transcript expression occurs (highest to lowest) in the pancreas, followed by the liver, placenta, lungs, kidneys, brain, ovaries, lymph nodes, spleen, heart, leukocytes and colon [80]. The transfection of HEK293 cells with the *ABCC10* gene confers resistance to various anticancer drugs including docetaxel, PTX, VCR, VLB, cytarabine, gemcitabine, 2',3'-dideoxycytidine, 9-(2-phosphonyl methoxyethyl)adenine (PMEA); epothilone B [77,78,81].

#### 3.1.3. ABCG2/Breast Cancer Resistance Protein (BCRP)/Mitoxantrone Resistant Protein (MXR)

The ABCG2 protein is a 72-kDa protein [82]. It is the first known half transporter with one TMD and one NBD to mediate MDR [83,84]. It is active upon homodimerization or oligomerization with itself or other transporters [44,50,85,86]. ABCG2 is a widely distributed transporter that is present mainly in the plasma membrane, and is highly expressed in the placental syncytiotrophoblasts, apical surface of small intestines, colon epithelium, liver canalicular membrane, luminal surfaces of microvessel endothelium of human brain and in the veins and capillaries of blood vessels [83,87,88,89]. Its wide distribution and expression suggests that it is involved in protecting the fetus and adult against toxins and xenobiotics [90]. ABCG2 is also abundantly expressed in the placenta and is also called ABCP (ABC transporter expressed in placenta) [91]. It is also expressed in colon cancer cell line S1-M1-80 that is resistant to MX, thereby giving ABCG2 the name MXR [92].

The substrates of ABCG2 include organic anion conjugates, nucleoside analogs, organic dyes, TKIs, anthracyclines, camptothecin-derived indolocarbazole topoisomerase I inhibitors, methotrexate and flavopiridols [44]. The ABCG2 transporter is a mediator of MDR in various cancers such as breast, colon, gastric, small cell lung, ovarian, gastric, intestinal cancers and melanomas [38,85,93]. In breast and ovarian cancer cell lines, epidermal growth factor can induce the activation of ABCG2 gene transcription. Mutations in the ABCG2 gene produce distinct substrate preferences for the mutant and wild-type variants. For example, a mutation at position 482 is the most important mutation for the determination of substrate specificity [94]. The amino acid arginine (Arg or R) is located on the carboxy terminal of the third transmembrane segment of the membrane-spanning domain, where substrate binding occurs probably due to the formation of salt bridges [44]. These mutations cause conformational changes and alter the drug binding and efflux capacity of the transporter [95,96,97]. Replacing Arg with threonine (Thr or T) or glycine (Gly or G) at position 482 produces changes in the substrate profiles among the variants [44,85]. Rhodamine 123, daunorubicin, and lyso-tracker green are substrates for mutant Gly and Thr variants, although they are not substrates for the wild type ABCG2 [44,98,99]. There are also some drugs, including MX, BODIPY prazosin, and many nucleoside inhibitors that are substrates of both wild-type and mutant ABCG2 [85].

## 4. Role of ABC Drug Transporters in the Development of Resistance to TKIs

Resistance to TKIs has been well documented, as they have been used progressively in clinical studies and administered to patients [100]. Point mutations within the kinase domain that cause the kinase less sensitive to TKIs are the most documented and analyzed mechanism of resistance to TKIs. As a result of these mutations, the TKIs are restricted from entering into the ATP-binding loop/activation loop of the kinase, causing resistance to TKIs. Apart from mutations in TKs domain there are other still not well-documented mechanisms that may contribute to TKIs clinical resistance. Resistance due to the expression of ABC drug transporters is one such mechanism, which has also been described for constitutive and acquired drug resistance to TKIs [101]. Many reports proposed that the ABCB1/Pgp/MDR1, ABCC1/MRP1 transporters and especially ABCG2/BCRP, play a significant role in development of resistance to TKIs. Some TKIs, including imatinib [102,103], nilotinib [103,104], dasatinib [105], gefitinib [106], danusertib [107], and canertinib [108] have been documented to be competitive or high-affinity substrates of ABCG2/BCRP and to interact with this transporter at its substrate-binding sites. ABCG2/BCRP increases the efflux of these TKIs from cancer cells, therefore causing resistance. Some studies showed that ABCB1/P-gp/MDR1 also conferred resistance to imatinib [109,110], nibotinib [111], and dasatinib [105]. Furthermore, Czyzewski and Styczynski [112] demonstrated that imatinib was a substrate for ABCC1/MRP1 and that it could increase the expression of ABCC1/MRP1, which played a role in imatinib resistance in CML. Lately, Shibayama *et al.* [113] proposed that sorafenib was a substrate for ABCC2/MRP2, which might contribute to sorafenib resistance. These findings indicate that overexpression of ABC transporters should be considered an important role in developing resistance to TKIs [114].

### Impact of ABC Transporters on the Pharmacokinetics and Toxicity of TKIs

ABC transporters are not only associated with MDR but also have a substantial impact on the pharmacokinetics and toxicity of TKIs. ABC transporters specifically, ABCB1, ABCC1 and ABCG2 are expressed not only in tumor tissues but also in normal tissues, such as the liver, kidneys, gastrointestinal tract, and blood-brain barrier. Therefore, these transporters can affect the pharmacokinetics (absorption, distribution, metabolism, and excretion) and toxicity of various antineoplastic agents, including TKIs, by inhibiting their intestinal uptake and brain penetration or by facilitating their elimination. Marchetti *et al*. [115] described that erlotinib was transported effectively by ABCB1 and ABCG2, demonstrating that the bioavailability of erlotinib after oral administration (5 mg/kg) was significantly enhanced in Bcrp1-/-/Mdr1a/1b-/- triple-knockout mice compared to wild-type mice. The brain distribution of gefitinib and dasatinib is detected to be limited by active efflux mediated by ABCB1 and ABCG2 [116,117]. Yang *et al.* [118] reported that tandutinib (MLN518) is a substrate of ABCB1 and ABCG2, impacting its oral absorption, systemic clearance, and brain penetration in rodents. These findings suggest that potent inhibitors of ABCB1 and ABCG2 may improve the bioavailability and efficacy of TKIs and hence may have direct clinical implications in cancer treatment with TKIs.

## 5. The ABC Transporter Mediated MDR Modulated by Tyrosine Kinase Inhibitors

MDR associated ABC transporters mediate resistance to TKIs by enhancing the efflux of substrate TKIs. Remarkably, numerous studies showed that, at clinically accomplishable concentrations, some TKIs could block these transporters and inhibit their drug efflux function, thereby reversing MDR to conventional chemotherapeutic drugs in cancer cells [6,38,119]. The use of TKIs to inhibit ABC drug transporter mediated-MDR in patients is a promising approach for treating drug resistant cancers. A detailed description of the reversal of ABC transporter mediated MDR by individual TKIs is reviewed here.

**Figure 2:**
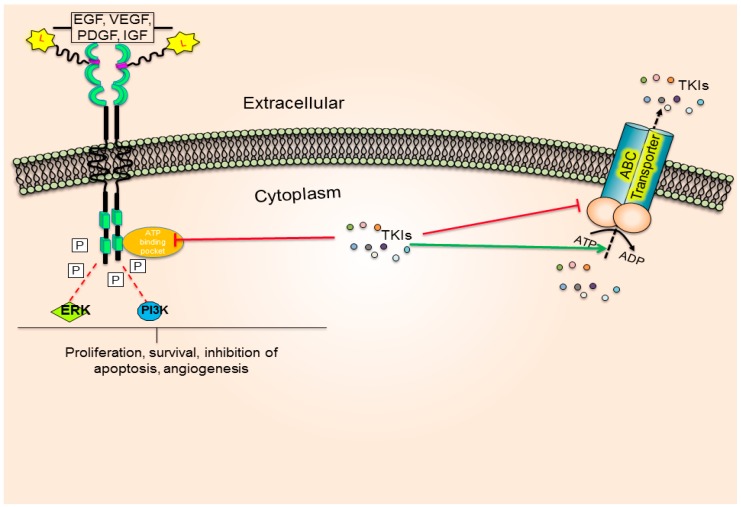
Schematic representation of TKI interaction with TK and ABC drug transporters.

A TKI blocks the ATP-binding pocket of RTK (Figure 2) and prevents the downstream phosphorylation event, thereby inhibiting cell proliferation and survival. Contrastingly, some TKIs also bind to the substrate-binding pocket of the transporter and are effluxed by the energy dependent hydrolysis of ATP molecules, thereby reducing the intracellular TKI concentration.

### 5.1. BCR- ABL Tyrosine Kinase Inhibitor

#### 5.1.1. Imatinib Mesylate (Gleevec^®^)

**Figure 3:**
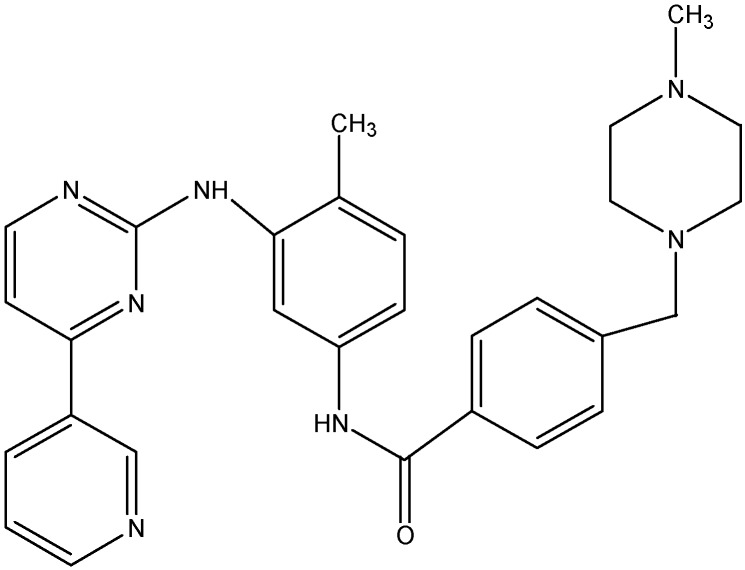
Structure of imatinib.

Imatinib (see Figure 3), approved by the FDA in 2001, is the first generation BCR-ABL inhibitor that inhibits proliferation of myeloid cells containing the BCR-ABL oncogene and is used in patients with chronic myeloid leukemia (CML). In addition to its use in CML, imatinib also plays a role in gastrointestinal stromal tumor (GIST) formation as it also inhibits the c-KIT and PDGFR tyrosine kinases [120]. Mukai *et al.* [42] reported that 2.5 μM imatinib could reverse resistance to VCR, PTX, etoposide, and actinomycin D in an epidermal carcinoma cell line (KB-G2) that overexpresses ABCB1. Another study described that imatinib potently reverses ABCG2-mediated resistance to topotecan and 7-ethyl-10-hydroxycamptothecin (SN-38). They reported that imatinib inhibits the substrate efflux function of ABCG2 and increase the accumulation of topotecan in cells expressing functional ABCG2 [121]. In addition, two independent studies reported that imatinib also reverses ABCC1 [122] and ABCC10 [123] mediated MDR. Shukla *et al.* reported that imatinib interacts with ABCB1 and ABCG2, at the transport-substrate site(s) and showed that imatinib competed for [^125^I]--Iodoarylazidoprazosin (IAAP) binding (a transport substrate of ABCB1 and ABCG2) to ABCB1and ABCG2, while it did not compete for the binding of [α-32P]-8-Azido-ATP, an ATP analog to either ABCB1 or ABCG2 [124]. Furthermore, they also showed that imatinib stimulate the ATPase activity in both ABCB1 and ABCG2 cells. These results indicate that imatinib acts like substrates for both ABCB1 and ABCG2.

#### 5.1.2. Nilotinib (AMN107)

**Figure 4:**
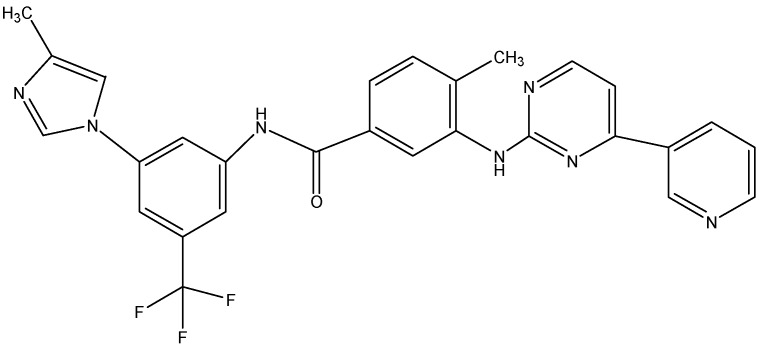
Structure of nilotinib.

Nilotinib (see Figure 4), regarded as second second-generation tyrosine kinase inhibitor, was developed with an aim to overcome imatinib-resistant CML.We were first to report that nilotinib has inhibitory effect on the efflux function of ABCB1 and ABCG2 [93]. We further described that nilotinib enhances the intracellular accumulation of PTX in cell lines overexpressing ABCB1 and MX in cells transfected with ABCG2. In addition to ABCB1 and ABCG2 nilotinib also reverses the ABCC10-mediated MDR and increases the intracellular accumulation of PTX and VCR (substrates of ABCC10) in ABCC10-transfected HEK293 cells [123]. Shukla *et al.* recently reported that nilotinib is a substrate of ABCG2 and it directly interacts with ABCG2 at the substrate binding sites, as it competes with the binding of [^125^I]-IAAP and also stimulates the transporter’s ATPase activity. They further suggested that ABCG2 might play an important role in nilotinib resistance [125]. Hiwase *et al.* showed that inhibition of ABCB1 activity by nilotinib can be used to enhance the intracellular concentration of dasatinib in CML cells, suggesting that a combination of low-dose dasatinib and nilotinib may provide an additive/synergistic anti-leukemic effect in leukemic stem cells that expresses ABCB1 and are refractory to TKI therapy [126]. In addition to this, nilotinib significantly potentiates the anti cancer activity of PTX in ABCB1, ABCC10 and DOX in ABCG2 tumor xenograft model [127].

#### 5.1.3. Dasatinib (Sprycel^®^)

**Figure 5:**
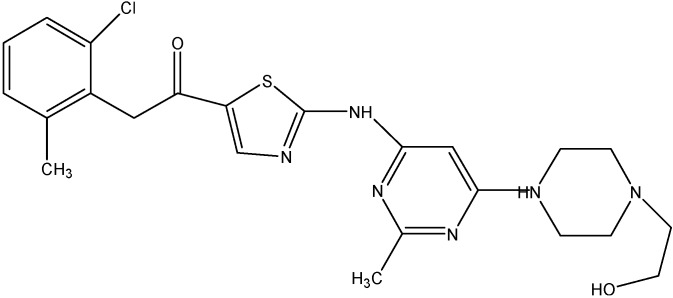
Structure of dasatinib.

Dasatnib (see Figure 5), another second-generation tyrosine kinase inhibitor, is a substrate of both ABCB1 and ABCG2 [128]. Recent reports suggest that dasatinib’s transport across the blood brain barrier is mediated by ABCB1 and ABCG2 and this uptake is significantly increased in the absence of ABCB1 and ABCG2 [117]. The involvement of these transporters in developing resistance to TKIs can be predicted by the substrate or inhibitory functions of nilotinib, imatinib, and dasatinib at high concentrations. Consequently, imatinib, nilotinib and dasatinib at concentrations of 4 μM, 2 μM, and 100 nM, respectively, have been reported to inhibit ABC transporter function.

#### 5.1.4. Ponatinib (Iclusig^®^)

**Figure 6:**
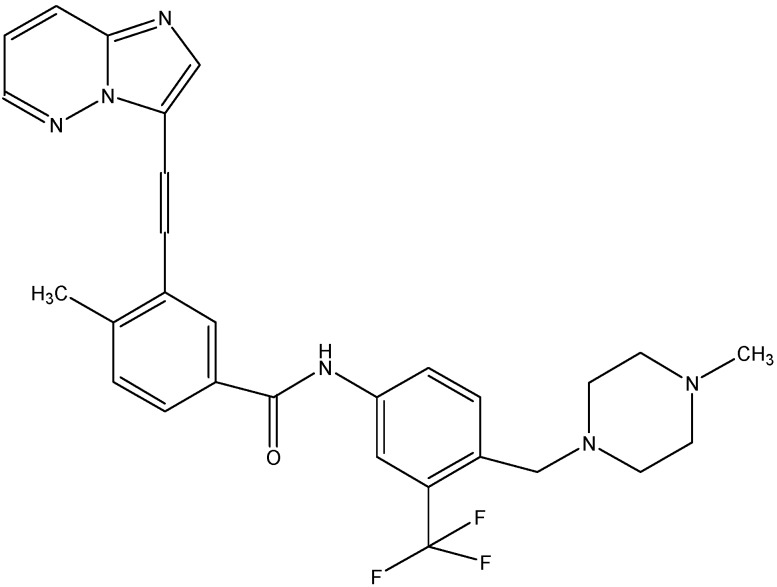
Structure of ponatinib.

Ponatinib (see Figure 6), a novel inhibitor of the BCR-ABL oncogene and Fms-like tyrosine kinase (FLT3), has been reported to inhibit the ABCB1, ABCG2 and ABCC10 transport function. Sen *et al.* studied the interaction of ponatinib with ABC transporters such as ABCB1, ABCC1 and ABCG2 [129]. Ponatinib showed an increased uptake of substrates of ABCG2 and ABCB1, but not ABCC1, in cells overexpressing these proteins, with a greater effect on ABCG2 than on ABCB1. Ponatinib inhibited [^125^I]-IAAP binding to ABCG2 and ABCB1, indicating its binding to the drug substrate sites, with IC_50_ of 0.04 μM and 0.63 μM, respectively. Ponatinib’s concentration dependent stimulation of the ATPase activity of both ABCB1 and ABCG2, suggests it to be a substrate of both the transporters. Very recently, another study done in our lab revealed that ponatinib also increases the uptake of ABCC10 substrates in HEK293/ABCC10 cells [130].

### 5.2. Epidermal Growth Factor Receptor Tyrosine Kinase Inhibitors

#### 5.2.1. AST1306

**Figure 7:**
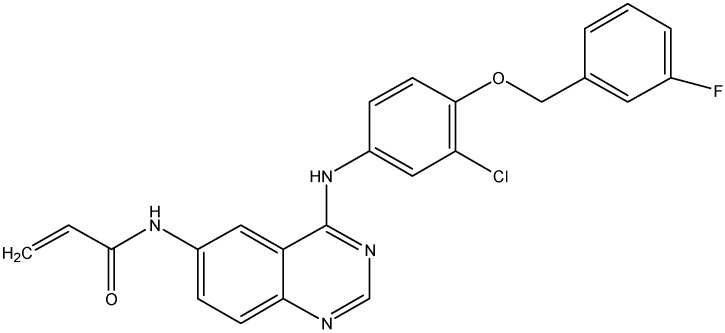
Structure of AST1306.

AST1306 (see Figure 7), an inhibitor of EGFR and ErB2, has been tested for its effect on the MDR induced by ABCG2. It is found to increase the cytotoxicity of MX and SN-38 in ABCG2 overexpressing cells and increase the intracellular accumulation of [^3^H]-MX in both ABCG2 expressing wild-type and mutant cells [131]. Additionally, AST1306 stimulated the ATPase activity of ABCG2 and its reversal of ABCG2 mediated MDR is more potent than lapatinib and erlotinib [131].

#### 5.2.2. Gefitinib (Iressa^®^)

**Figure 8:**
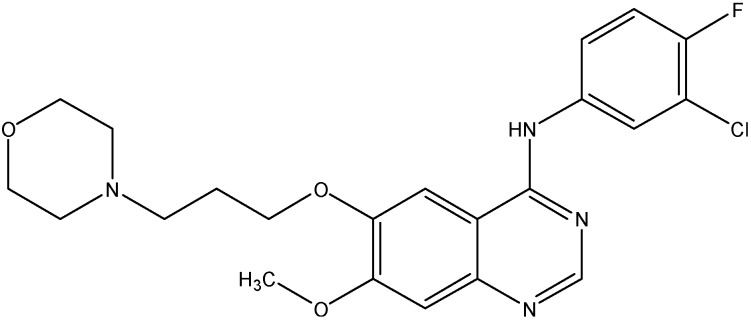
Structure of gefitinib.

Gefitinib (see Figure 8), a selective EGFR (ErbB1) tyrosine kinase inhibitor has been approved for the treatment of non-small cell lung cancer.It prevents autophosphorylaton of EGFR resulting in the inhibition of cyclin- dependent kinase activity and cell cycle arrest in the G_1_ phase [132]. Previous studies suggest that gefitinib can inhibit transport function of ABCG2 and reverse resistance to SN-38 in ABCG2 overexpressing cells [133]. Gefitinib treatment enhanced the oral bioavailability of irinotecan in mice after simultaneous oral administration in both *in vitro* and *in vivo* studies. Kitazaki *et al.* in 2005 reported that gefitinib reversed the resistance caused by ABCB1 to PTX and docetaxel in ABCB1 overexpressing PC-6 PTX lung cancer and MCF-7/Adr breast cancer cells and also increases the intracellular accumulation of rhodamine-123 in ABCB1 overexpressing cells [134].

#### 5.2.3. Erlotinib (OSI-774; Tarceva^®^)

**Figure 9:**
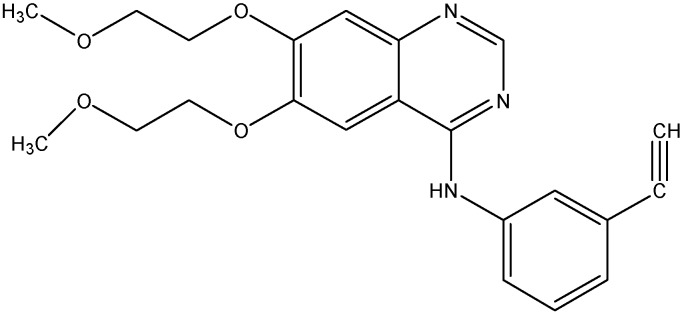
Structure of erlotinib.

Erlotinib (see Figure 9) is a selective ErbB1 inhibitor that inhibits EGFR dependent cellular proliferation and causes cell cycle arrest at G_1_ phase [135]. Earlier studies have shown that erlotinib reverses the ABCB1 and ABCG2 mediated MDR and also increases the accumulation of [^3^H]-PTX in HEK293/ABCC10 cells [50,72]. The phase II trial in patients with bronchoalveolar carcinoma showed an improved sensitivity to erlotinib. Erlotinib in combination with trastuzumab showed better tolerability and enhanced antitumor activity [136].

#### 5.2.4. Lapatinib (GW-572016)

**Figure 10:**
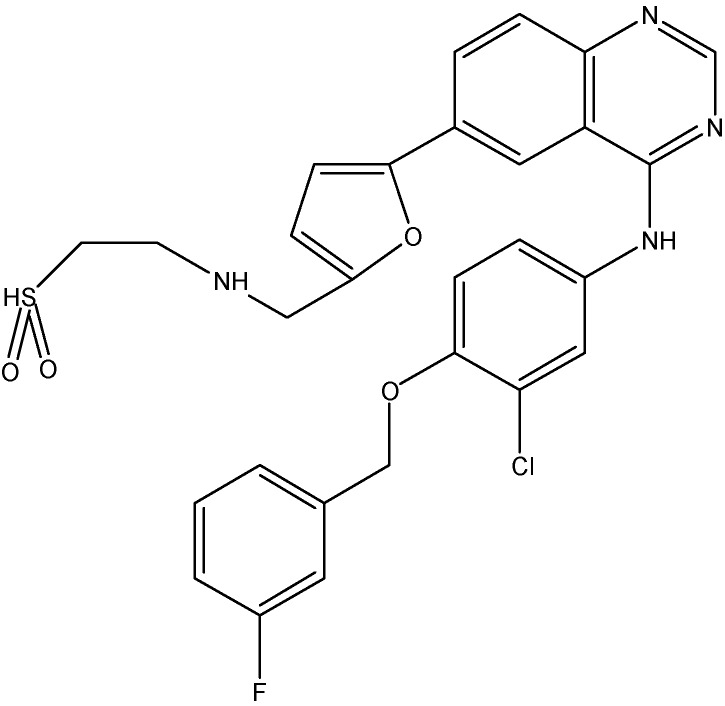
Structure of lapatinib.

Lapatinib (see Figure 10) is a non-selective EGFR inhibitor that inhibits both ErbB1 and ErbB2. Studies done in the past have shown that lapatinib increases the accumulation of DX or MX in ABCB1 or ABCG2 overexpressing cells and enhanced the ATPase activity of both ABCB1 and ABCG2 [137]. It also, increases the chemosensitivity of PTX in ABCB1 overexpressing tumor xenograft models [137]. In addition to this, lapatinib has also been shown to reduce ABCC10 mediated MDR and to increase sensitivity of *ABCC10*-transfected HEK293 cells to docetaxel, PTX, VLB, and vinorelbine [72]. Thus, lapatinib reverses the multi-drug resistance caused by ABCB1, ABCG2, and ABCC10 by inhibiting their transport function.

#### 5.2.5. Canertinib (CI-1033)

**Figure 11:**
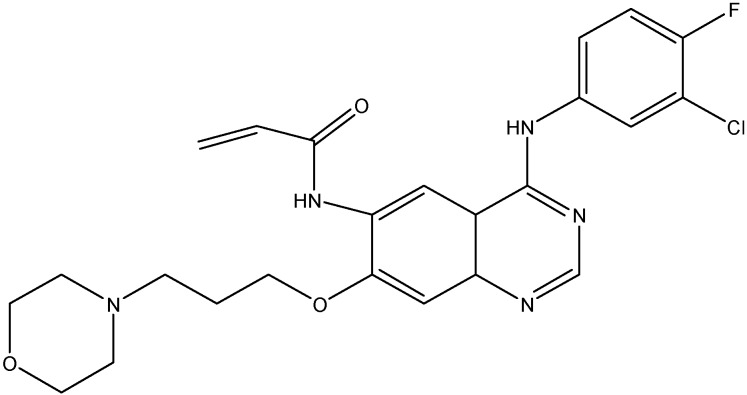
Structure of canertinib.

Canertinib (see Figure 11) is a non-selective EGFR inhibitor that inhibits EGFR kinase activity. In addition to its inhibitory effect on ErbB1 and ErbB2, canertinib also shows some activity against ErbB3 and ErbB4 as well [138]. Erlichman *et al.* reported that canertinib increases the cytotoxic effects of SN-38 and topotecan by enhancing their intracellular accumulation in cells overexpressing ABCG2 [108]. For its effect on ABCB1, canertinib was shown to increase the accumulation of pazopanib, a substrate for ABCB1, in ABCB1 overexpressing MDCKII cells and thus blocks the efflux function of ABCB1 [139]. Furthermore, when administered with erlotinib, canertinib is found to produce a 2–2.5 fold increase in brain disposition of pazopanib [139].

#### 5.2.6. Icotinib

**Figure 12:**
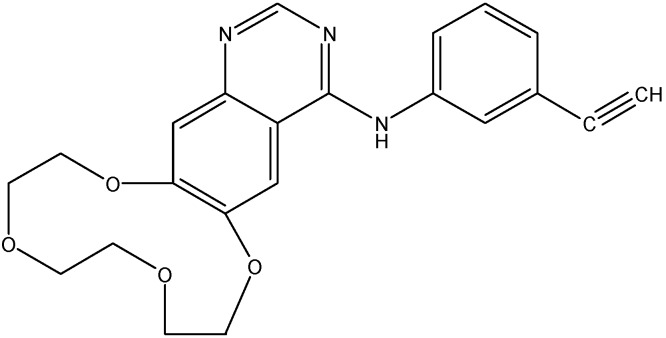
Structure of icotinib.

Icotinib (see Figure 12) is an EGFR inhibitor, which has been shown to be active against NSCLC [140]. Recently, it was reported that icotinib reverses ABCG2 transporter mediated MDR in H460/MX20 cell lines. It increases the intracellular accumulation of [^3^H]-MX and inhibits its efflux as well. Additionally, icotinib stimulates the ATPase activity and inhibits the photo labeling of ABCG2, which indicates that icotinib interacts with drug binding pocket. Furthermore, icotinib significantly enhance the anticancer activity of topotecan against H460/MX20 tumor xenografts [141].

### 5.3. Vascular Endothelial Growth Factor Tyrosine Kinase Inhibitors

#### 5.3.1. Telatinib

**Figure 13:**
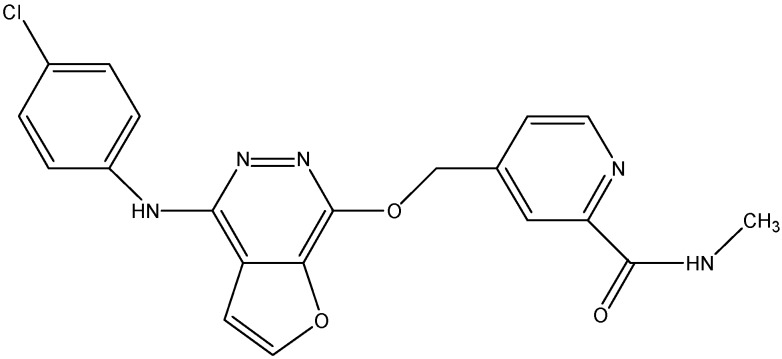
Structure of telatinib.

Telatinib (see Figure 13) is an orally available tyrosine kinase inhibitor of VEGFR-2, VEGFR-3 PDGFR-β and cKIT. A study done in our laboratory has shown the inhibitory effects of telatinib on the ABCG2 efflux transporter. At 1 μM, telatinib increased the intracellular accumulation of [^3^H]-MX and reduced its efflux from ABCG2 overexpressing cells at the same concentration [142]. Furthermore, telatinib stimulated the ATPase activity and also in combination with DX, it decreased the ABCG2 overexpressing tumor size [142].

#### 5.3.2. Sunitinib (SU11248)

**Figure 14:**
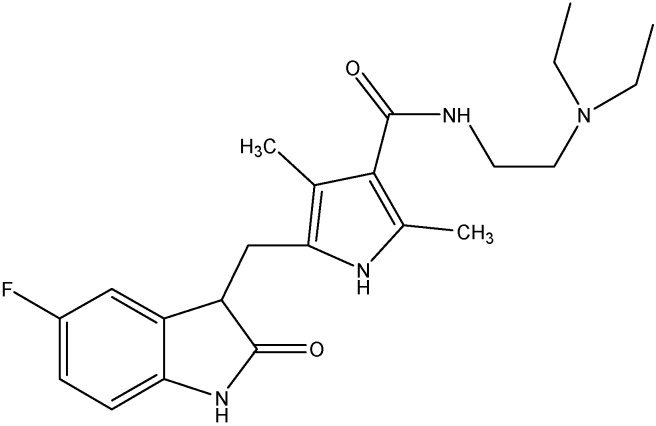
Structure of sunitinb.

Sunitinib (see Figure 14), a broad spectrum tyrosine kinase inhibitor inhibits VEGFR, c-KIT, PDGFR, and FLT-3. It is reported that sunitinib is able to inhibit the activity of ABCB1 and ABCG2 and reverse the resistance mediated by these transporters [96,143]. Results of clinical trials suggest the antiangiogenic activity of sunitinib (Phase I) and its use in patients with metastatic kidney cancer (Phase II) [144].

#### 5.3.3. Sorafenib (BAY 43-9006)

**Figure 15:**
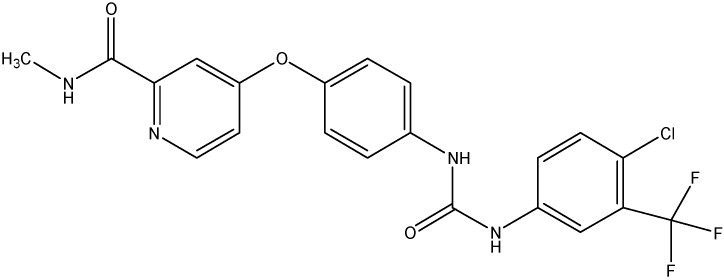
Structure of sorafenib.

Sorafenib (see Figure 15), a novel Raf kinase inhibitor, inhibits a broad spectrum of tyrosine kinases including the VEGFR, EGFR, and PDGFR kinases [145]. Owing to this broad-spectrum inhibition of sorafenib, it shows significant activity against colon, lung, renal and pancreatic tumors. Sorafenib’s effect on the ABC transporters has been studied in the past and it was found that sorafenib inhibited the efflux of cytarabine (a substrate of ABCC10 and ABCC11) [146].

#### 5.3.4. Motesanib

**Figure 16:**
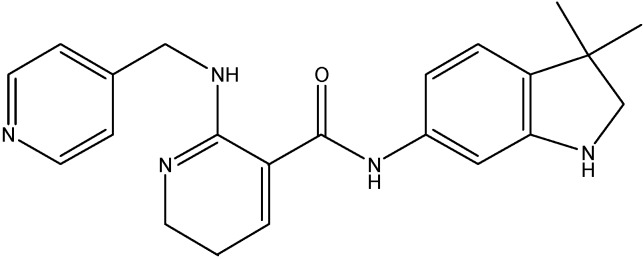
Structure of motesanib.

Motesanib (see Figure 16), an inhibitor of VEGFR-1, VEGFR-2, VEGFR-3, and PDGFR, has been shown to completely inhibit the ABCB1 efflux function and partially reverse the ABCG2-mediated MDR. Wang *et al.* reported that motesanib increases the intracellular accumulation of [^3^H]-PTX in cells overexpressing ABCB1 transporter and stimulates the ATPase activity of ABCB1 as well. However, the results of western blotting showed that it had no effect on the ABCB1 protein levels, after treatment with motesanib for 72 h. Furthermore, motesanib at 3 μM is found to increase the sensitivity to MX for cells overexpressing ABCG2 transport [147].

### 5.4. Platelet- Derived Growth Factor Inhibitors

#### 5.4.1. Masitinib

**Figure 17:**
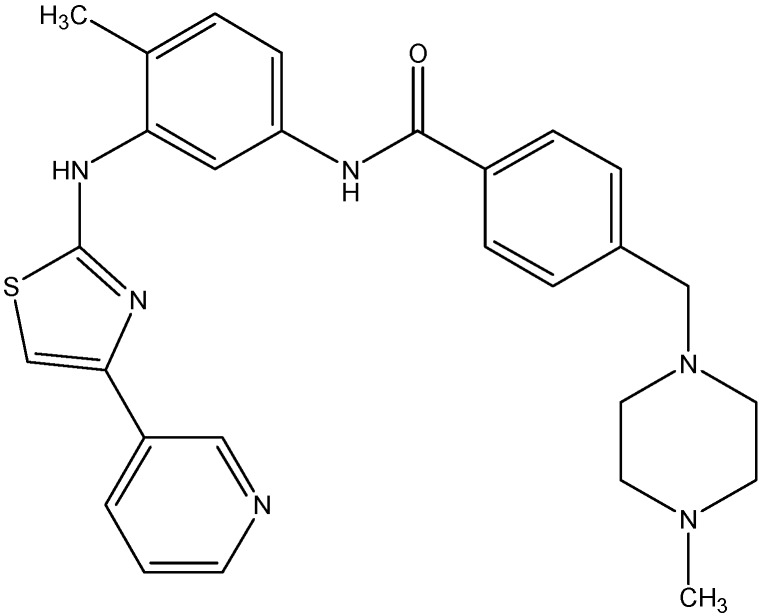
Structure of masitinib.

Masitinib, a phenylaminothiazole derivative (see Figure 17), is an inhibitor of c-Kit and PDGFR α &β. Recent studies done in our laboratory have demonstrated the inhibition of the ABCC10 and ABCG2 transport function by masitinib [148,149]. Masitinib was found to increase the intracellular accumulation of PTX at 2.5 μmol/L and also to inhibit the growth of ABCC10-expressing tumors in combination with PTX [148]. For its effect on ABCG2, masitinib was found to decrease the resistance to MX, SN-38 and DX in HEK293 and H460 cells overexpressing ABCG2, at 1.25 and 2.5 μM [150]. Furthermore, the intracellular accumulation of [^3^H]-MX was found to be increased by masitinib thereby inhibiting the function of ABCG2 [149].

#### 5.4.2. Linsitinib

**Figure 18:**
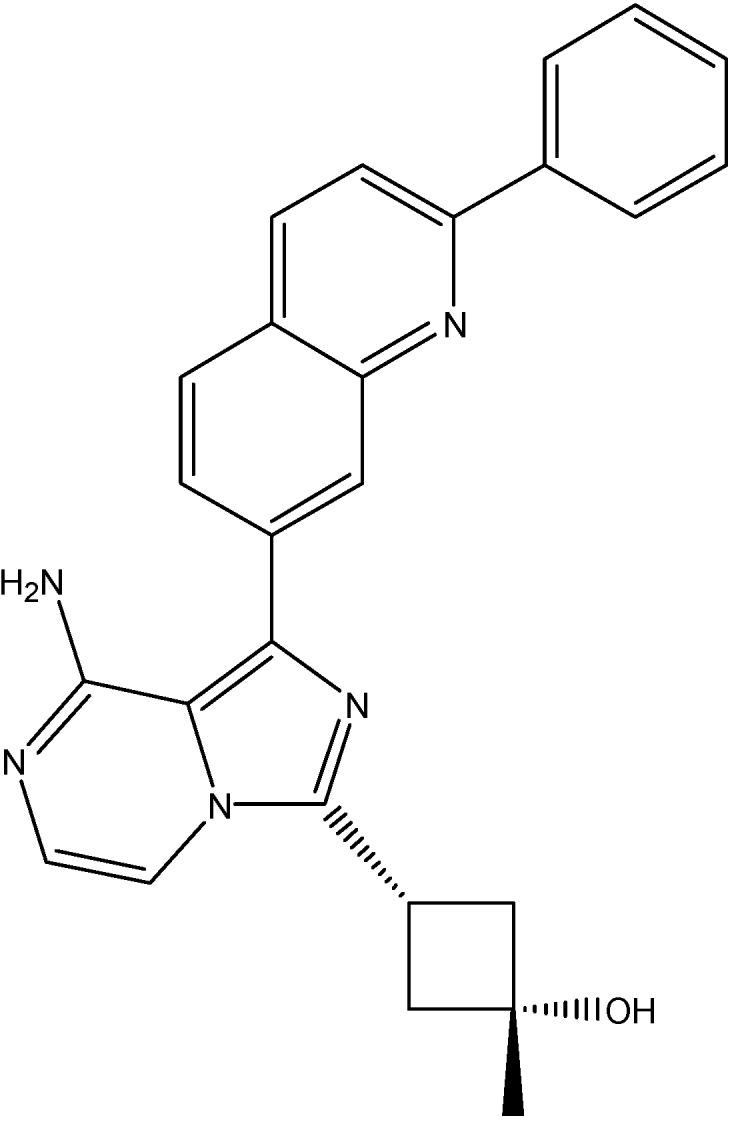
Structure of linsitinib.

Linsitinib (see Figure 18), an insulin-like growth factor 1 (IGF-1R)/insulin receptor (IR) kinase inhibitor It is reported to be an ABCG2 and ABCC10 mediated MDR inhibitor. It increases the effects of MX and SN-38 in ABCG2-overexpressing cells and stimulated the ATPase activity of ABCG2 in a concentration dependent manner [150]. In addition to its effect on ABCG2, linsitinib also increases the effects of PTX, docetaxel and VLB in ABCC10- overexpressing cells and increased the intracellular accumulation of [^3^H]-PTX in ABCC10-overexpressing cells [150]. On the other hand, its effect on ABCB1’s substrate cytotoxicity is moderate and there is no significant alteration in the cytotoxicity of the substrates of ABCC1 [150].

### 5.5. Fibroblast Growth Factor Receptor Inhibitors

#### PD173074

**Figure 19:**
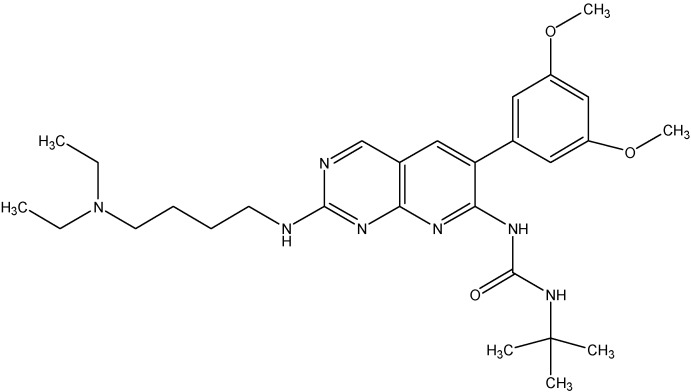
Structure of PD173074.

PD173074 (see Figure 19), a pyrido[2,3-d]pyrimidine was synthesized based on the crystal structure of FGF2-inhibitor complex and was seen to exhibit a high degree of complementarity towards the tyrosine kinase domain of FGFR1. PD173074 showed promising results both *in vitro* and *in vivo* to block the growth of small cell lung cancer (SCLC). Recently, we reported that PD173074 reverses the ABCB1 and ABCC10 mediated multidrug resistance [151,152]. PD173074 significantly increases the concentrations of PTX in ABCB1 overexpressing and ABCC10 transfected cells. In addition, PD173074 stimulated the ATPase activity of ABCB1 in a concentration-dependent manner, suggesting a direct interaction with the transporter. Interestingly, PD173074 did not inhibit photo labeling of ABCB1 with [^125^I]-IAAP, showing that it binds at a site different from that of IAAP in the drug-binding pocket [151].

### 5.6. B-Raf/MEK/ERK Pathway Inhibitors

#### Vemurafenib (PLX 4032)

**Figure 20:**
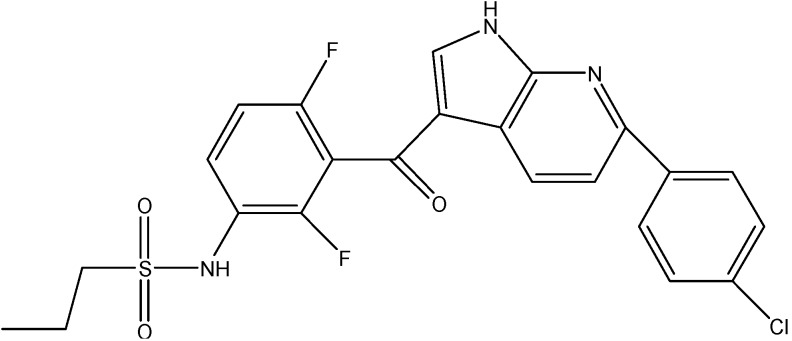
Structure of vemurafenib.

Vemurafenib (see Figure 20), a B-Raf inhibitor is used for treatment of metastatic melanomas [153]. Recently it has been reported to inhibit the ABCB1, ABCC10 and ABCG2 mediated MDR. It increases the intracellular accumulation of PTX in cells overexpressing ABCB1 and ABCC10 and MX in cells over expressing ABCG2 transporter. Furthermore, vemurafenib at 20 μM did not alter the expression level of ABCB1, ABCC10, and ABCG2 when treated up to 96 h [154].

**Table 1:** Tyrosine Kinase Inhibitors and their association with ABC drug transporters.

Inhibitor	Tyrosine Kinase Target	Neoplasm(s) Targeted	Clinical Status	Association with ABC Transporters
Imatinib (Gleevec, Glivec)	BCR-ABL, c-KIT and PDGFR	CML and ALL, MDS/MPD, ASM HES, CEL, DFSP and GIST	Approved, 2001	ABCB1 [122], ABCC1 [122], ABCG2 [121] and ABCC10 [123]
Nilotinib (Tasigna)	BCR-ABL	CML	Approved, 2007	ABCB1 [93], ABCG2 [93] and ABCC10 [123]
Dasatinib (Sprycel)	BCR-ABL and Src	CML, ALL	Approved, 2006	ABCB1 [117] and ABCG2 [128]
Gefitinib (Iressa)	EGFR	NSCLC	Approved, 2006	ABCB1 [134] and ABCG2 [133]
Erlotinib (Tarceva)	EGFR andHER2	NSCLC	Approved, 2004	ABCB1, ABCG2 and ABCC10 [50,72]
Lapatinib (Tyverb)	EGFR, HER2	HER2-positive breast cancer	Approved, 2007	ABCB1, ABCG2 [137] and ABCC10 [72]
Canertinib (CI- 1033)	EGFR, HER2 and ErbB- 4	Metastatic breast cancer.	Phase II	ABCB1 [108] and ABCG2 [139]
Icotinib	EGFR	NSCLC	Approved in china, 2011	ABCG2 [141]
AST1306	EGFRandHER2	Solid tumors	Phase I	ABCG2 [131]
Sorafenib (BAY 43-9006)	VEGFR, EGFR	Renal and pancreatic cancer	Phase II	ABCC10 and ABCC11 [146]
Motesanib	PDGFR, VEGFR and c-KIT	NSCLC, GIST, breast cancer.	Phase III	ABCB1 and ABCG2 [147]
Sunitinib (Sutent)	FLT3, PDGFR, VEGFR and c-KIT	RCC, GIST	Approved, 2006	ABCB1 and ABCG2 [96,143]
Telatinib	VEGFR-2, VEGFR-3 PDGFR-β and cKIT	Colorectal and gastric cancer	Phase II/III	ABCG2 [142]
Masitinib	c-Kit ,PDGFR α, β	Metastatic gastrointestinal stromal tumors	Phase II/III	ABCG2 and ABCC10 [148,149]
Linsitinib	IGF-1R/IR	Adrenocortical carcinoma	Phase III	ABCG2 and ABCC10 [150]
Ponatinib	FGFR1-4, FLT3 and PDGFR	CML and Ph+ ALL	Approved, 2012/suspended for vascular clots 2013	ABCB1, ABCG2 [129] and ABCC10 [130]
PD173074	FGFR	NSCLC	Phase I	ABCB1 [151] and ABCC10 [152]
Vemurafenib	B-Raf/MEK/ERK	Melanona	Approved, 2011	ABCB1, ABCG2 and ABCC10 [154]

BCR-ABL: Breakpoint cluster region-Abelson complex; c-KIT: Mast/stem cell growth factor receptor Kit; PDGFR: Platelet-derived growth factor receptor; Src: Proto-oncogene tyrosine-protein kinase Src; EGFR: Epidermal growth factor receptor; HER2: Human epidermal growth factor receptor 2; VEGFR: Vascular endothelial growth factor receptor; MEK: Mitogen-activated protein kinase; ERK: Extracellular signal-regulated kinase; FLT3: Fms-like tyrosine kinase 3; IGF-1R: Insulin-like growth factor 1 receptor; IR: Insulin receptor; ALK: Anaplastic lymphoma kinase; c-MET: Hepatocyte growth factor receptor; FGFR: Fibroblast growth factor receptor; CML: Ph+ chronic myeloid leukaemia; ALL: lymphoblastic leukaemia; MDS/MPD: Myelo displasic syndrome-myeloproliferative disorder; ASM: Aggressive systemic mastocytosis; HES: Hyper eosinophilic syndrome; CES: Chronic eosinophilic leukaemia; DFSP: Dermato-fibro sarcoma protuberan.

## 6. Adverse Cutaneous Reactions of Tyrosine Kinase Inhibitors

Adverse effects of TKIs are very low, when compared to the traditional anti neoplastic agents. However, several studies reported that use of TKIs associated with several systemic side effects. Apart from systemic side effects, they are highly associated with muco-cutaneous side effects [155]. Studies revealed that TKIs such as imatinib, nilotinib and dasatinib are immensely associated with these side effects. The degree of cutaneous side effects associated with these three drugs includes skin rashes, puritus, superficial edema and hyper pigmentation or hypo pigmentation. The severity of side effects mostly related to their dose and mechanism behind these side effects related to TKIs pharmacological action. For example, superficial edema associated with imatinib is due its pharmacological inhibition of PDGFR, a growth factor responsible for interstitial fluid homeostasis [127,156]. Another study reported that increased photosensitivity associated with use of imatinib is due to inhibition of ABCG2 mediated phyrin transport. The authors also postulated that polymorphisms or ABCG2 inhibition by imatinib result in enhanced photosensitivity [157]. Patients taking TKIs should be medically managed by early detection and symptomatic treatment of cutaneous side effects [155].

## 7. Conclusions and Therapeutic Perspective

Based on the established studies, TKIs act as both substrates and inhibitors of ABC drug transporters, depending on the concentration of the inhibitor used and its affinity to the transporter. Typically TKIs at lower concentrations depicts substrate-like properties but at higher concentrations inhibit the function of the transporter. As a result, the dosage regimen, bioavailability of TKI, its affinity to ABC transporters and expression levels of these transporters in tumor cells can be critical factors in clinical resistance to TKIs. These can ultimately determine the ability of ABC transporters to confer resistance to TKIs in patients. However, majority of research in the field of drug resistance to TKIs is predominantly focused on mutations in the tyrosine kinase domain, while ABC transporter mediated TKI resistance remains a challenge. This should be examined as a substitute mechanism by which tumor cells circumvent the cytotoxic effects of TKIs. On the other hand, some TKIs are found to inhibit ABC transporters, thereby increasing the concentration of substrate anticancer agents *in vitro* and *in vivo*. The strategy of using these TKIs as adjuvants in chemotherapy especially for drug-resistant tumors, which overexpress ABC drug transporters, is promising. However, it is critical to find the selective TKI to selective transporter with the right dosing, which would allow minimal pharmacokinetic interaction of the chemotherapeutic drugs and maximal specific inhibition of the transporter in the target tumor. Taken all this into account, an ideal TKI would be an agent that can inhibit the activity of the target TK without being transported by ABC drug transporters and. Thus, new synthetic and pre-clinical research should focus on developing such molecules. This will provide development of novel potent TKIs with high selectivity, without associated resistance due to abundance of ABC drug transporters.
